# Measuring Green Exposure Levels in Communities of Different Economic Levels at Different Completion Periods: Through the Lens of Social Equity

**DOI:** 10.3390/ijerph19159611

**Published:** 2022-08-04

**Authors:** Qinyu Cui, Yiting Huang, Guang Yang, Yu Chen

**Affiliations:** 1 School of Architecture and Urban Planning, Shenzhen University, Shenzhen 518060, China; 2 Urban Planning, Shenzhen New Land Tool Planning & Architectural Design Co., Shenzhen 518172, China

**Keywords:** green justice, housing price, the age of communities, community green space, street view images, green space accessibility, Shenzhen

## Abstract

Exposure to green spaces contributes to residents’ physical and mental health and well-being. The equitable allocation of green space has also become an increasingly important issue for society and the government. This study takes 3281 communities in Shenzhen as the analysis units. Using web crawlers, semantic segmentation based on deep learning, web map path planning and entropy weighting methods, four types of residents’ daily green exposure indicators are calculated, including community green space ratio, green view index (GVI), park accessibility, and the weighted composite green exposure index. The results reveal inequalities in the level of green exposure in Shenzhen’s communities across economic classes, mainly in GVI and comprehensive green exposure. We also found that the level of composite green exposure is relatively stable; however, green space ratio attainment levels for newer communities are increasing and GVI and park accessibility attainment levels are decreasing. Finally, among the newly built communities: compared to the low-income level communities, the high-income level communities have a significant advantage in green space, but the mid-income level communities do not have such an advantage. The main findings of this study can provide policy implications for urban green space planning, including the need to prioritize the addition of public green space near older communities with poor levels of green exposure, the addition of street greenery near communities with poor levels of composite green exposure, and ensuring that parks have entrances in all four directions as far as possible.

## 1. Introduction

In the context of Chinese cities, one of the most common concerns is socio-economic inequality. Indeed, studies reveal that China has shifted from a relative doctrine of equality to a society marked by increasing economic disparity since the policy of the socialist-oriented market economy [1].

The public resources represented by green spaces can increase housing prices in the surrounding communities [2,3,4]. Especially after the 1998 housing reform characterized by privatization and marketization, residents with similar class characteristics, as evidenced by their income and consumption capacity, tend to cluster in specific urban spaces under the filtering mechanism of house prices, generating residential differentiation [5,6,7]. The phenomenon of residential differentiation raises concerns about the equitable access to public resources, including green space, across multiple socio-economic statuses, because of the particular importance of community green exposure for the health of residents [6].

This article attempts to quantify the equity of community green exposure. The potential mechanism of human exposure to green space has been referred to as green exposure [8,9,10]. We chose Shenzhen as a case study, and community green space ratio, green street visibility, park accessibility, and a weighted comprehensive of the three indicators to evaluate the equity of green exposure in communities of different economic levels. Then, the communities were divided into six categories according to the period when they were built-up to explore the development trend of green exposure levels in communities of different economic levels. Finally, the significance of the differences in green exposure levels for communities of different economic classes within the same built-up period was tested by the chi-square test. The results of this study provide important insights for future municipal decisions on how to accurately allocate urban green space facilities.

## 2. Literature Review

Labib et al., based on the definition of green exposure provided in the existing studies, classified the concept of green exposure into availability, visibility, and accessibility [11]. Firstly, availability, also called provision, means the amount of available green space in a community, including the communities’ internal green space ratio [12,13] and green coverage [14]. It is the most straightforward and most easily quantifiable indicator and is commonly used in urban studies where the community is the unit of analysis. Secondly, visibility, also known as the “green view ratio [15]”, refers to the proportion of greenery in the human view and is mainly measured by the Green View Index (GVI). Finally, accessibility is usually operationalized as the distance between residents and large green areas [16,17]; large green areas mainly refer to city parks and community parks. Green exposure due to availability, green exposure due to visibility, and green exposure due to accessibility vary significantly [11].

Green space is a common generalized term used to describe natural areas in urban environments, such as parks, community gardens, and public green spaces [18,19,20]. The content of green exposure can correspond to residents’ three most frequent exposures to green space: community green spaces, street greenery, and urban parks. We have summarized the roles of the three green spaces, including each common and featured role (Table 1). Generally, the benefits of green spaces for residents are manifold.

In previous green exposure assessment studies, the green space objects are mainly parks, and few studies capture the problems of finer-scale green space [27]. In fact, visual sources provide 90% of the information that humans acquire in their environment [36]. Green spaces within communities and their surrounding streets are those that most residents come into contact with most frequently daily [26]. Especially during the restricted activity radius during COVID-19, exposure to and viewing green spaces plays an important role in alleviating residents’ negative emotions and enhancing their mental health [27,37,38].

Early on, urban green land ratios and per capita green areas were used to reflect the spatial construction level of urban green space in different regions; later, urban green space was studied for spatial equity and social equity [39]. Urban parks, as a public good supported by public funds, have been the main object of current green justice research. Studies on green justice represented by parks show the characteristic of (green space) as the backyard of the rich, and the underprivileged at the bottom show a clear green space disadvantage [40,41,42]. However, parks cannot represent all ranges of green space objects, and in recent years studies on green equity have begun to enrich the types of green space objects. Wu et al. found that green space inequity was mitigated when urban parks and street greening were considered in the meantime in a green justice study [43]. In another green justice study, community green spaces were added. It was assumed that all communities would be open, and people could access public urban parks and other communities’ green spaces, but green space inequalities have not been improved [12]. These studies demonstrate the potential for green justice research by enrichment of green space objects.

Access to green space is a significant environmental justice issue. It means that everyone has the right to be protected from specific environmental issues (such as pollution and climate change) and to obtain standard services (green spaces) [44]. Green space inequality indicates differences in the accessibility or available quantity of green space, which is related to residents’ race [45], income [46], and age [47].

Mladenka et al. use empirical evidence from Chicago to show that socio-economic class may be a determining factor in the distribution of green space [48]. Numerous studies in China show residential socio-spatial heterogeneity and that residents’ choices of urban-dwelling may represent their socio-demographic position in urban China [5,6,7,49]. Some scholars frequently use data from the estate’s secondary market (a market formed when commercial properties are first traded into circulation) to explain the organization of urban residential space [7] and map differences in economic status and class features among numerous purchasing groups [6,16,17].

As technology continues to evolve, there are breakthroughs in acquiring community built-up time data and quantifying new types of green data. Firstly, obtaining community built-up time data published by developers on real estate transaction websites allows for a comparative assessment of green exposure in communities with different built-up periods. Community green space ratio from real estate websites can also support fine-scale assessment of green space within communities [50,51]. Secondly, the GVI calculation is usually achieved by semantic segmentation methods with the help of open-source data (e.g., Google Street View, Tencent Street View, Baidu Street View) and deep learning techniques [52]. Finally, web maps can improve the accuracy of accessibility calculations compared to traditional ArcGIS platforms for calculating road network distances [53], because open maps on the Internet can consider complex scenarios such as underpasses, overpasses, and traffic lights, making the calculation results infinitely close to the real ones.

In summary, most existing green justice studies focus on park-oriented green objects, ignoring the fine-scale greenery that community residents are more frequently exposed to [12,27,43]. Meanwhile, current studies lack comparisons from a time-based perspective [6]. It is difficult to interpret the real situation of community green equity comprehensively, and the trends of green exposure changes in built communities at different periods are ignored.

In other words, the latest data and methods are bringing fresh research possibilities to the fore. Our study incorporates green equity measures into more green data and introduces a time-based perspective to evaluate many types of green exposure levels in communities of different economic levels. This could help to accurately assess community green space equity and propose more precise green strategies.

## 3. Materials and Methods

### 3.1. Study Area

Shenzhen is near Hong Kong and is part of the Pearl River Delta. Shenzhen has become one of China’s most populous and inventive cities since the Special Economic Zone was established in 1979. Although Shenzhen’s urban spatial structure is polycentric, there is a clear difference in urban development between the north and the south, based on the central boundaries of the city. At the end of 2020, the city covered an area of 1995 km^2^, 980 km^2^ of which were built-up areas, and the city’s resident population had reached 17.6 million; with the city’s built-up area greening coverage rate of 43.38%, and a built-up area green space rate of 37.36%, the greening indicators ranked among the top in the country [54]. Hundreds of parks of various types were established by the end of 2021, compared to only five parks at the start of the reform and opening-up (Late 1978).

**Figure 1:**
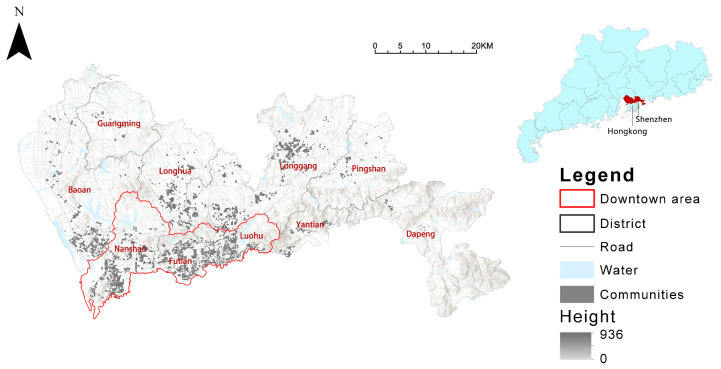
Study area.

The city is divided into ten administrative districts, as shown in Figure 1. Shenzhen’s downtown areas, mainly located in Nanshan, Futian, and Luohu districts, concentrate a large number of jobs; the outside districts have also developed gradually in recent years, generating a large amount of residential space and several sub-central areas.

### 3.2. Data Source

#### 3.2.1. Community Data

1. Data access.

Communities’ data were captured on the FANGTIANXIA website (https://sz.fang.com/ (accessed on 30 March 2021)) by the web crawler, which is one of the top three trading platforms for online housing in China. It was the first to be established and covers the largest data and number of website hits. The community data was gathered using the crawler technique, which included particular location, price, community kind, community age, and green space rate. Ultimately, after data clearance, we obtained information on 3281 observations in Shenzhen.

**Figure 2:**
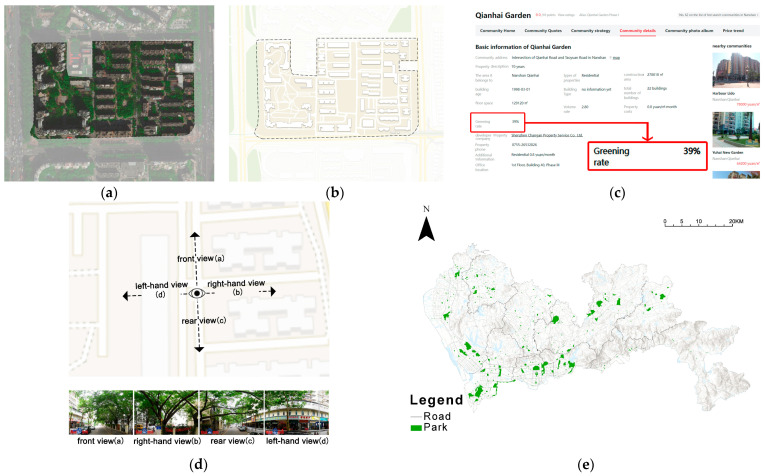
Data sources:(**a**,**b**) Amal’s green spaces in the communities, (**c**) a page on the housing website (FANGTIANXIA) showing community information, which includes the community green space ratio, (**d**) green spaces in Baidu Street—view images, (**e**) park from Baidu Map.

Next, the community locations were geocoded (relying on Baidu Maps) to obtain each community’s latitude and longitude coordinate information. The residential points were projected onto a base map in ArcGIS. Using the Baidu Maps API (Application Programming Interface), a search was made by latitude and longitude of each community to obtain the actual boundaries of each community (Figure 2).

2. Community economic classification.

3281 communities were divided into five equal numbers of partitions [16,17]. Then, community prices in the brackets 80% and more, more than 20% and less than 80%, and up to 20% could represent high-income, mid-income, and low-income levels, respectively. Because of the high housing costs in Shenzhen, purchasers may spend a considerable amount of their income on the house [12,55,56]. Therefore, housing prices can be considered as an important factor reflecting people’s income and socio-economic position [12,55,56].

3. Community built-up period classification.

The particular year of housing reform, 1998, characterized by privatization and marketization, was used as the dividing point [12,16], followed by a 5-year cycle of grouping communities. On the one hand, this is because Chinese planning programs are based on a 5-year interval; on the other hand, it is because the number of communities built in some years is less and needs to be aggregated. The community was divided into six periods according to the time of completion: 1980 to 1993, 1994 to 1998, 1999 to 2003, 2004 to 2008, 2009 to 2013, and 2014 to present.

The community green space ratio is almost unchanged since the community was built, while the street greenery and parks (amount) are generally getting better all the time. Note that, due to the time limitation of street view images (SVIs) and park data acquisition, we only assessed the community green space by the current state of urban green space. Communities of different built-up periods were then grouped to compare their green exposure level.

#### 3.2.2. Green Space Data

Green space data was collected in and around communities, including green spaces within the community, green spaces in streets around the community, and urban parks.

1. Community green space.

Figure 2a,b show that community green spaces are excluded from the commercial map service. Fine-scale community green space data can be obtained directly from the FANGTIANXIA website, which provides a register of completed communities, including their green space rates (Figure 2c). We also took a sample of 100 (approximately 3%) for manual verification of the accuracy of community green space rates. Only two sources of community data were highly different from the actual situation, and almost all (98%) of the data were accurate.

2. Street greenery.

The vegetation in the street view images collected using the Baidu Map API is depicted in Figure 2d (accessed on 5 July 2021, in Chinese only). First, each processed road segment is separated into 50-m-distance points based on the OSM (OpenStreetMap) road network, and the coordinates of each point data are recorded. The Baidu Map API was used to download four street view photos of each coordinate along the route and in the direction of the perpendicular road using the coordinates of 321,548 points. In the end, 1,286,192 images were collected in the research area.

3. Park.

As shown in Figure 2e, park data was obtained from Baidu Map using web crawler technology (accessed on 10 August 2021). Based on the criteria for community parks in Shenzhen, parks of 1–5 ha were identified as community parks; parks of 5 ha or more were considered as comprehensive parks. Meanwhile, green spaces that charge admission, such as botanical gardens and golf courses, were not included [17]. After checking with the official government-published parks, 99 municipal parks and 294 community parks in Shenzhen were included in this study.

### 3.3. Green Indicator Calculation

#### 3.3.1. Community Green Spaces

Community green space data is obtained directly from real estate websites and reflects the ratio of community green space to the total community area.

#### 3.3.2. Street Greenery

The visibility metric represents the green space that community residents can see and perceive on the street. This study defines the resident activity area as the Euclidean buffer zone of 100 m around the community boundary outwards (excluding the community itself). The reason for choosing this distance is that, according to the *Shenzhen Urban Planning Standards and Guidelines (2013)*, the width of urban roads in Shenzhen is 12 to 80 m. Therefore, a 100 m buffer can cover the sampling points around the community.

**Figure 3:**
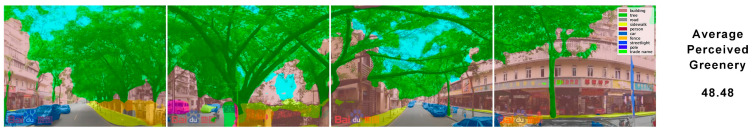
Segmentation results for street view images.

The pyramid scene parsing network (PSPNet) is a pixel-level object detection and classification method that generates solid semantic segmentation results, with pixel-level accuracy of more than 80.2% [57]. It has been applied by several prior studies [54,55,56] to extract streetscape features such as trees, sky, and building views from SVIs to inform the urban environment [58], housing prices [50], and urban design [59]. We used PSPNet to extract the pixel ratios of individual features as view indices from SVIs (144, 810 images), and more than 30 elements were detected (Figure 3). Perceived greenery can be defined as urban greenery’s overall visual contact intensity in a live scene. The formula is as follows:

(1) $$GVI_{j}=\frac{\sum_{i=1}^{4}Pic_{greenry}}{\sum_{i=1}^{4}Pic}\times 100\%$$

where $GVI_{j}$ is the green view rate value of the jth sample; the denominator represents the total area of the streetscape corresponding to each sample point (including the four horizontal views to the east, south, west, and north), and the numerator represents the green area covered by the panoramic segmentation.

Finally, each community’s GVI is the average of the GVI values of all street points within the community’s 100 m boundary buffer. Each community has an average of 45 street points within its buffer zone, with a minimum of 10 points.

#### 3.3.3. Park Accessibility

**Figure 4:**
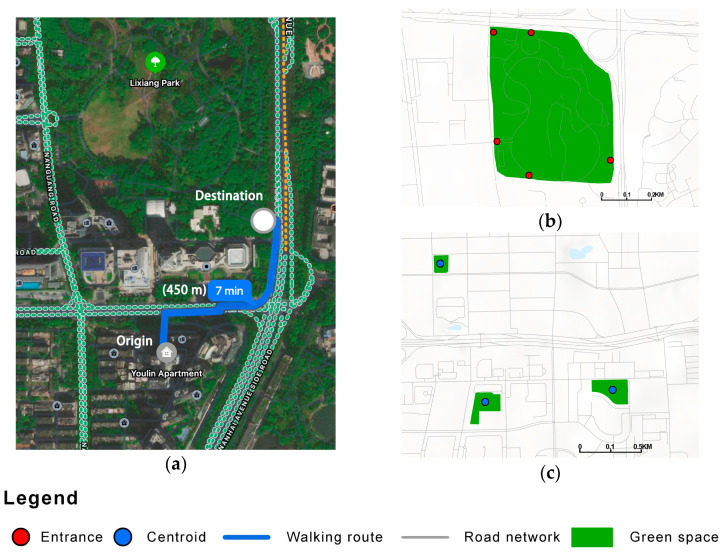
Walking path in the Amap navigation route. (**a**) origin, destination, and distance and travel time in a specific case. (**b**) Destinations of urban green spaces at the municipal level. (**c**) Destinations of urban green spaces at the community level.

Accessibility indicators describe the distance from the community to the nearest park. As the location of households in each community varies, when regarding departure points, using the community center point as the departure point can be considered the average location for all community residents to depart from. Regarding destinations, we defined the centroid and entrances of green spaces as destinations according to the level of the green space [17,60,61]. The destinations of community parks (area < 5 ha) and municipal parks (area ≥ 5 ha) were established as the centroids and entrances, respectively (Figure 4b,c).

Amap is a web map similar to Google Navigation [53,62]. A matrix from the origin to the destination is created, and the Amap API’s path planning method is then used to measure the accessibility of the green space. We sent batch requests for path planning to the Amap API server to calculate the walking path distance from each community to the nearest park (Figure 4a (accessed on 7 February 2022)).

#### 3.3.4. Green Level Standards

Considering that different green spaces have different contact standards, we produced an evaluation standard through the normative standards of different green spaces. First, drawing on China’s residential area design code, *Standard for urban residential area planning and design (GB50180-2018)*, a 30% green space rate was selected as the benchmark for community green space attainment. Next, the 10 min (800 m) walking distance was selected as the benchmark for park accessibility, based on the *Spatial planning guidance to the community life unit (TD/T1062-2021)*. Finally, there is no national-level evaluation standard for street greening in China. Referring to the official document issued by the Ministry of Land, Infrastructure, Transport and Tourism, which is responsible for urban construction in Japan [63], a 25% greening rate was selected as the benchmark for street greening.

Using standardized and entropy methods to calculate the comprehensive green evaluation index of each community, which is comprehensive in order to consider the green exposure level of the community, the calculation steps are as in Formulas (2) to (5):

1. Standardization of green indicators.

The level of community green exposure varies considerably under the same green indicator. To measure more the difference between the level of green exposure and the ideal standard (Section 3.3.4), this study uses the baseline value of the indicator for quantification.

Positive indicator:

(2) $$Y_{i}=\left\{\begin{array}{r} 1,\;\;\;x_{i}\geq s_{i}\\ x_{i}/s_{i},\;\;x_{i}<s_{i} \end{array}\right.$$

Inverse indicator:

(3) $$Y_{i}=\left\{\begin{array}{r} 1,\;\;\;x_{i}\leq s_{i}\\ x_{i}/s_{i},\;\;x_{i}>s_{i} \end{array}\right.$$

where $x_{i}$ is the measured value of the ith sample; $s_{i}$ is the baseline value of the ith sample; and $Y_{i}$ is the standardized value of the ith sample (0 ≤ $Y_{i}$ ≤ 1).

2. Determination of green indicator weights.

(4) $$H_{i}=-\frac{1}{\ln(n)}\overset{n}{\underset{j=1}{\sum }}P_{ij}\ln\left(P_{ij}\right)$$

(5) $$W_{i}=\frac{1-H_{i}}{\sum_{j=1}^{n}(1-H_{i)}}$$

where $H_{i}$ is the entropy value of the ith sample, and $P_{ij}$ is the proportion of the total number of green indicators in the jth community for the ith sample; $W_{i}$ is the entropy weight of the ith sample; *n* is the total number of elements.

### 3.4. Chi-Square Test

The chi-square test is a very versatile hypothesis test for counting data, the purpose of which is to compare the degree of agreement or goodness of fit between the theoretical and actual frequencies. This method was used in this study to verify whether there was a significant difference in the level of green space compliance between high- or mid-income level communities and low-income level communities.

If the results of the chi-square test are not significant, the original hypothesis cannot be rejected. It indicates that high- or mid-income level communities tend to have no significant difference in the attainment level of green exposure from low-income level communities; conversely, if the results of the chi-square test are significant, the original hypothesis is rejected. It indicates that high- or mid-income level communities tend to differ significantly from low-income level communities in terms of their attainment level for green exposure.

### 3.5. Spatial Correlation Analysis

1. Finding areas with weak levels of community green exposure.

The local Getis-Ord Gi* index is a local spatial autocorrelation index based on a distance weight matrix that detects high-value and low-value aggregation. This study analyses the degree of community green exposure aggregation at the local spatial level. The calculation formula is as follows.

(6) $$G_{i}^{*}=\frac{\sum_{j=1}^{n}W_{ij}\left(d\right)X_{j}}{\sum_{j=1}^{n}X_{j}}\left(j\neq i\right)$$

where: $X_{j}\;$ is the element attribute value of the jth spatial unit; *n* is the total number of elements; $W_{ij}\;$ is the spatial neighbourhood weight matrix within distance  d. If the distance between the i th and j th spatial units lies within a given critical distance d, they are considered neighbours, and the element in the spatial weight matrix is 1. Otherwise, the element is 0. To distinguish between cold and hot spots in the horizontal spatial distribution of community green spaces, it is also necessary to normalize the $G_{i}^{*}\;$ index as follows.

(7) $$\;Z\left(G_{i}^{*}\right)=\frac{G_{i}^{*}-E\left(G_{i}^{*}\right)}{\sqrt{Var\left(G_{i}^{*}\right)}}$$

where: $E\left(G_{i}^{*}\right)$ is the mathematical expectation; $Var\left(G_{i}^{*}\right)$ is the coefficient of variation. When $Z\left(G_{i}^{*}\right)$ is positive and significant, it means that the values around spatial unit i are relatively large, i.e., the hot spot area; when $Z\left(G_{i}^{*}\right)$ is negative and significant, it means that the spatial clustering of low values of spatial unit  i, i.e., the cold spot area.

2. The spatial relationship between communities’ age and price and green exposure levels.

Using global bivariate Moran’s I, the spatial connection between community price and three forms of green exposure was studied. With the following computation steps (8) and (9), these analyses were used to examine spatial correlations across the research area [64]:

(8) $$I_{P,A}=\frac{N\sum_{i}^{n}\sum_{j\neq i}^{n}W_{ij}Z_{i}^{p}Z_{j}^{A}}{\left(N-1\right)\sum_{i}^{N}\sum_{j\neq i}^{N}W_{ij}}$$

(9) $$I^{\prime }{}_{P,A}=Z_{i}^{P}\overset{n}{\underset{j=1}{\sum }}W_{ij}Z_{j}^{A}$$

where $I_{P,A}$, and $I^{\prime }{}_{P,A}$ refer to the global and local bivariate Moran’s I, respectively, N refers to the total number of residential communities, $Z_{i}^{P}$ refers to the standardized value of housing price for the ith community, and $Z_{j}^{A}$ refers to the standardized value of green exposure level for the jth community. $W_{ij}$ represents the *N*-by-*N* Euclidean distance weighting matrix for the spatial correlation between ith and jth communities. In the current work, we calculated bivariate Moran’s I by using GeoDa 1.16. We used 999 permutations to assess the statistical significance of bivariate Moran’s I; the significance values were defined at <0.01.

The values of bivariate Moran’s I range from −1 to 1. For the bivariate Moran’s I with statistical significance, the positive and negative values indicate spatial aggregation (positive spatial correlation) and spatial fragmentation (negative spatial correlation), respectively [65]. The cluster map resulting from the local bivariate Moran’s I can determine four kinds of spatial correlations at the community level: High–High type refers to high-age communities encircled by the high green space ratio, and so on.

## 4. Results and Discussion

### 4.1. Overall Assessment of the Level of Community Green Exposure

#### 4.1.1. Overview Green Space ratio, GVI, and Accessibility

Table 2 shows the statistical results of the green space ratio, GVI, and park accessibility levels of 3281 communities in Shenzhen collected in this study. Of these, 76.0%, 72.2%, and 56.7% of communities met the normative standards for green space ratio, GVI, and accessibility, respectively; however, in terms of average benchmark values, GVI overtook green space ratio to rank first. 

**Figure 5:**
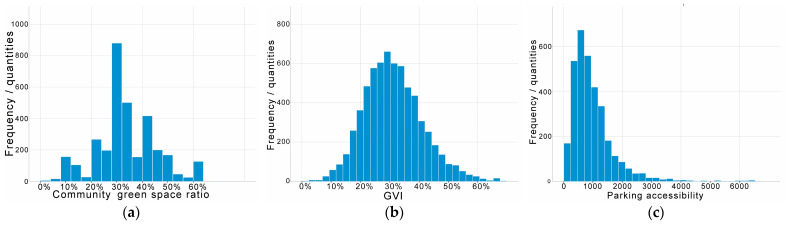
Levels of green exposure in 3281 communities in Shenzhen for (**a**) community green space ratio, (**b**) GVI, and (**c**) park accessibility.

Specifically, the structure of the data is shown in Figure 5. The coefficients of variation for green space ratio and GVI are not very different, at 0.347 and 0.318, respectively, which is the medium variation; the coefficient for park accessibility is 0.755, which is a high variation. The coefficient of variation reflects the degree of dispersion in the data distribution, and the medium and high variability of the green space indicators suggest that we need to examine the variability across locations further.

**Figure 6:**
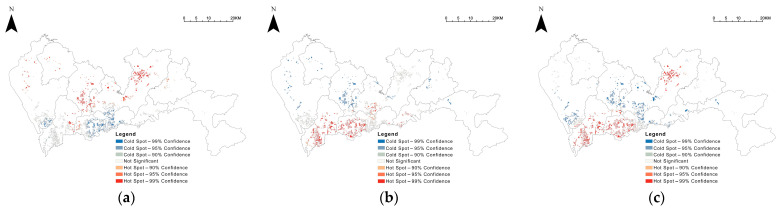
Hotspot analysis of community green exposure, in terms of (**a**) community green space ratio, (**b**) GVI, and (**c**) park accessibility.

The spatial distribution of green spaces ratio, GVI, and accessibility was analysed in hotspots (Getis-Ord Gi*), and the results were visualized (Figure 6). The Luohu and Futian districts within the city’s central area and the Bao’an central area to the west of the central area have lower community green space rates. In comparison, the non-central areas generally have higher community green space rates. The results of GVI and accessibility are similar. Central area communities have mostly higher GVI and accessibility, and non-central area communities are generally weaker; however, there are exceptions with higher park accessibility in the Longgang central area communities.

#### 4.1.2. Overview of Comprehensive Green Exposure Levels

**Figure 7:**
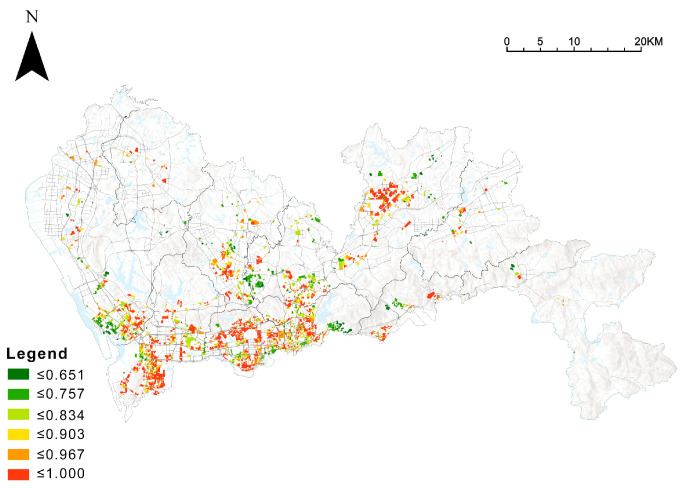
Spatial pattern of comprehensive community green exposure levels.

Using the baseline values of green space ratio, GVI, and accessibility, entropy values and weights were further calculated (Table 3). Finally, the comprehensive green exposure level was calculated for each community (Figure 7).

**Figure 8:**
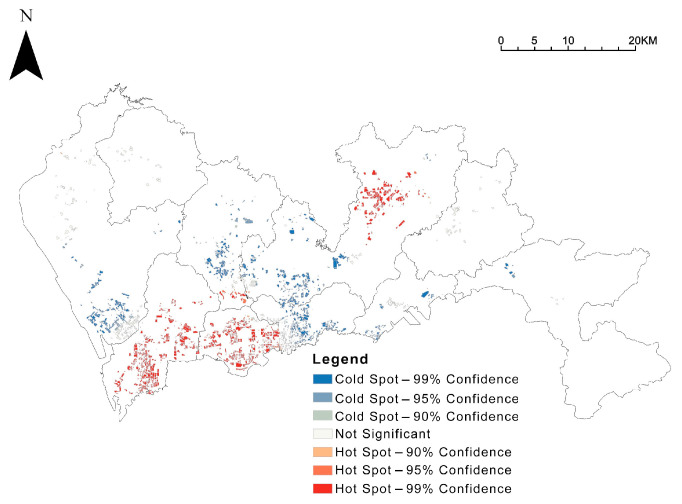
Hotspot analysis of comprehensive community green exposure levels.

Further analysis through the hotspot analysis (Figure 8) reveals that there is a structure of “two centers sandwiched by one belt” in the exposure level of Shenzhen’s integrated community green space, which is high in the center and low in the outside, but also high in the Longgang central area on the outside. The high-value communities are mainly located in Futian, Nanshan, and Longgang central areas. 

### 4.2. Analysis of the Level of Community Green Exposure for Different Economic Classes

Standard deviation ellipses are commonly used to describe the spatial characteristics of geographical distributions, such as trends in concentration, dispersion, and trends in the direction of distribution [66]. This study introduces the standard deviation ellipse to better analyze the spatial distribution of communities of different economic classes [67].

**Figure 9:**
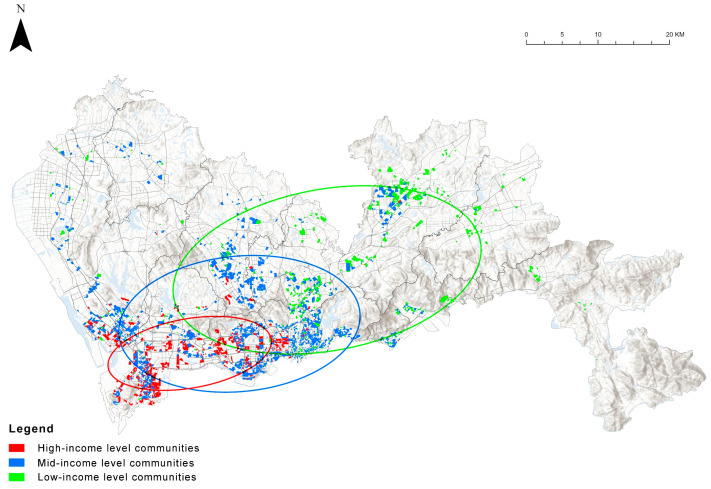
Spatial distribution of communities of different economic classes.

The spatial distribution of communities of different economic classes is shown in Figure 9. High-priced communities are mostly concentrated in downtown areas, such as Nanshan, Futian, and Luohu District, which are more clustered and less discrete, with a clear directional orientation. With the lowering of the economic strata, the distribution of communities begins to move towards the northeast and becomes more discrete.

**Figure 10:**
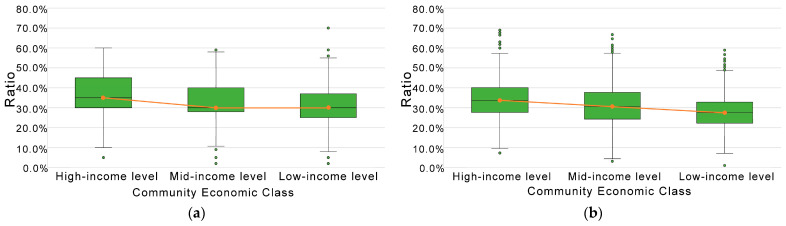

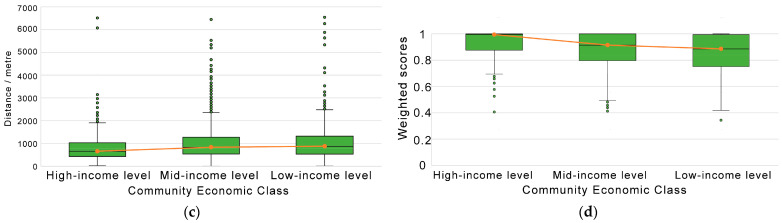
Comparison of green exposure levels in communities of different economic classes in terms of (**a**) community green space ratio, (**b**) GVI, (**c**) park accessibility, and (**d**) comprehensive green exposure.

The results of green space levels in communities of different economic classes are shown in Figure 10. In Shenzhen, the more expensive the community, the better the level of green space residents enjoy, whether overall or at the median level. That is, in Shenzhen’s communities, the levels of green space ratio, GVI, accessibility, and comprehensive green exposure are unevenly distributed among communities of various economic classes. However, the green space ratio and accessibility difference are not significant between mid-income level and low-income level communities.

**Figure 11:**
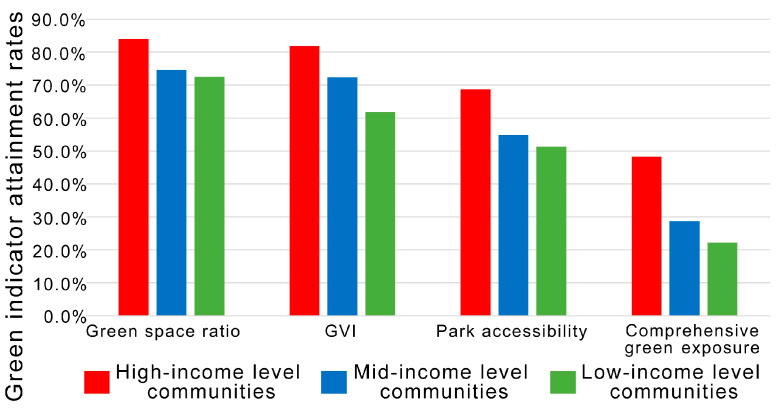
Attainment rates for green exposure levels in communities of different economic classes.

After the level of green exposure has met the specification, absolute equality is no longer sought. Therefore, we further counted the level of attainment rates for green exposure in communities of different economic levels, and the results are shown in Figure 11. High-income level communities continue to lead in the three green exposure attainment levels, with mid-income level communities second and low-income level communities last. Significantly, the attainment rate of the comprehensive green exposure level (where all three green space levels are attained simultaneously) shows more gaps across economic classes.

### 4.3. The Relationship between Community Prices and Green Exposure Levels

#### 4.3.1. Objective Differences in the Level of Community Green Exposure in Different Locations

The results of our study in Shenzhen show that communities in central urban areas benefit more from public green facilities (GVI and park accessibility) than those in non-central urban areas. However, the central urban areas of Luohu and Futian district have a poorer community green space ratio. In comparison, the non-central areas have a better community green space ratio. Differences in community public green space are attributed to Shenzhen’s spatial distribution of green space resources. In contrast, private green space differences are attributed to communities’ built-up period.

Firstly, we present an overview of the public green facilities. Table 4 shows that the Downtown District leads the Non-Downtown District in terms of the percentage of park space per unit area (about 4 times) and the percentage of parks quantity per unit area (about 2 times). In theory, communities in downtown areas have better accessibility to parks than non-downtown areas. Similarly, GVI reflects urban place quality [32,67]. Downtown areas take on functions such as city image display, and the overall street appearance of downtown areas is better than that of non-downtown areas. Communities in downtown areas also have better GVI of surrounding streets than non-downtown areas.

Next comes private greenery, where the age of the community heavily influences the level of community green space. *The Code of urban Residential Areas Planning & Design (GB50180-93)*, introduced in 1993 and implemented in 1994, began to emphasize the requirement for a green space ratio in communities, which means that the green space ratio in newly built communities should not be less than 30%. On the one hand, the earlier (pre-1994) built communities were not subject to this design standard; on the other hand, the later communities were mostly gated, and the concept of community green space began to take form and was gradually taken into account [67]. So the attainment rate of community green space was much lower before 1994 (57.70%) than post-1994 (78.28%). 

**Figure 12:**
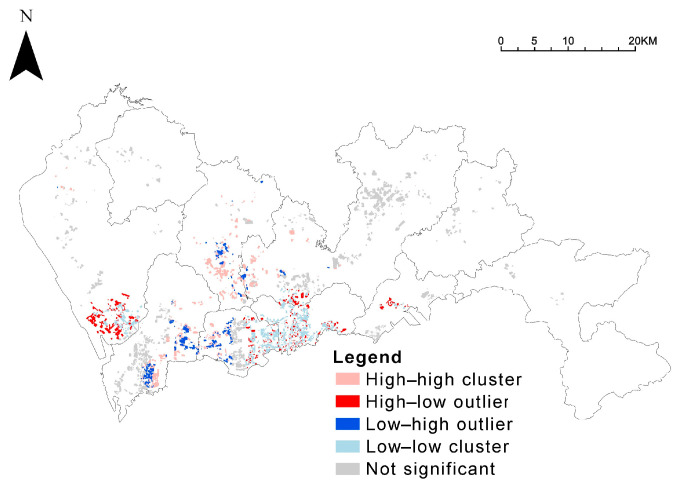
Spatial correlation between green space ratio and community age.

**Figure 13:**
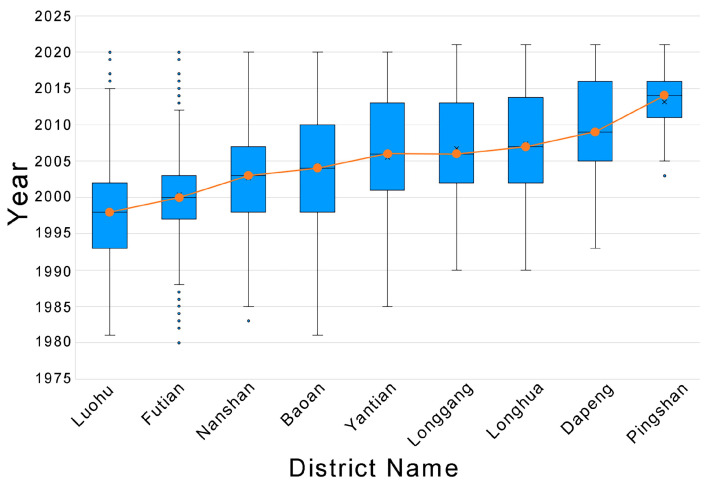
Built-up life of communities in different districts.

The result of global bivariate Moran’ I was 0.043 (*p* < 0.01), revealing a positive spatial correlation between year of community completion and green space rate. The relationship between community age and green space ratio is further shown in the clustering diagram of the local bivariate Moran’ I index (Figure 12), particularly for the Low–Low type (old built-up communities with low green space ratio). The number of Low–Low types is 856, the largest share of the four portfolio types. That is, an older community has lower green space ratios. The Low–Low type is mainly concentrated in the Luohu District, the earliest developed area in Shenzhen, and communities within the area were generally built earlier (Figure 13). Its early years were not governed by residential green space ratio norms, resulting in a low community green space ratio within the Luohu District. 

These communities need to be given focused attention and consideration to enhancing the public green spaces around them. However, the addition of public green space is very difficult, especially with the addition of parks. For older communities, it is a difficult issue to increase the level of green exposure.

#### 4.3.2. Differences in People’s Economic Capacity

**Figure 14:**
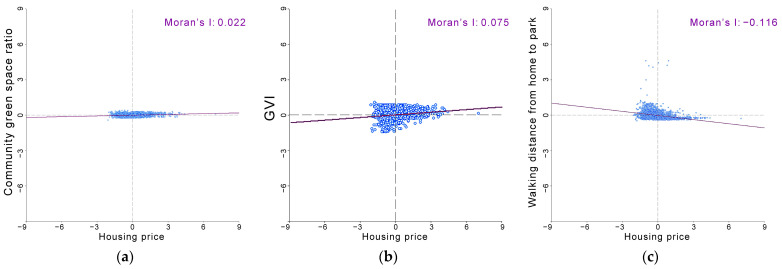
Correlation between green exposure level and community price, in terms of (**a**) community green space ratio, (**b**) GVI, and (**c**) park accessibility.

It has been shown that location is the most important factor influencing house prices [68]. The availability of green space drives the property value of the surrounding area [2,3,4]. After the housing market reform, urban residents purchase housing through the commercial housing exchange market according to their financial ability to meet their housing needs. The results of the bivariate Moran for green space, green view, and accessibility with house prices in this study were 0.022, 0.075, and −0.116 (*p* < 0.01), respectively (Figure 14), confirming that communities with higher housing prices perform better in terms of green exposure levels.

On the one hand, because of the location, the closer to the city center, the higher the cost of living in the community; on the other hand, the green space advantage can boost this cost. Communities in central areas with both locational and public green space advantages have extremely high prices while, the closer to the suburbs, the opposite conclusion is reached. In this case, the divergence in the economic ability of urban residents allows people with higher economic attributes to live in communities with better levels of greenery [69].

### 4.4. The Relationship between Community Age and Green Exposure Levels

**Figure 15:**
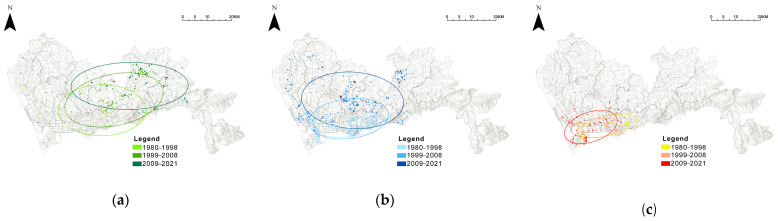
Spatial distribution of communities of different economic classes at different built-up periods, in terms of (**a**) low-income level communities, (**b**) mid-income level communities, and (**c**) high-income level communities.

The spatial distribution of communities of different economic classes at different built-up periods is shown in Figure 15. Over time as it was built, high-income level communities developed from the Luohu district to the west, clustering in the city center area in almost every period. Mid-income and low-income level communities tend to expand from the city center area to the suburbs, with the low-income level communities showing a greater tendency to expand in a north-easterly direction and the mid-income level communities showing a greater tendency to expand northwards.

#### 4.4.1. Trends in Levels of Community Green Exposure 

**Figure 16:**
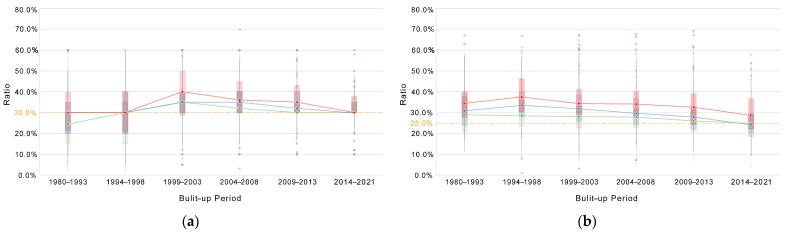

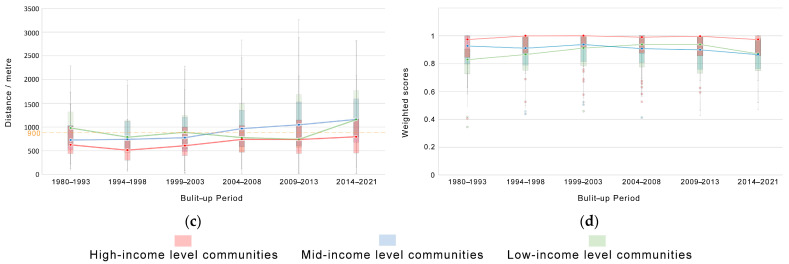
Comparison of green exposure levels in communities of different economic classes at different periods, in terms of (**a**) community green space ratio, (**b**) GVI, (**c**) park accessibility, and (**d**) comprehensive green exposure.

As shown in Figure 16a–c, the level of green space ratio in the community has increased and then decreased over time. However, since 1999, about 75% of communities have been able to meet the norm (greater than or equal to 30%); the level of GVI and accessibility has decreased. This result is somewhat surprising, against the background of ever-better material life, as the level of public green space in the newer built-up communities seems to be deteriorating. The trend in the level of three types of green space in communities of different economic classes is consistent, which also seems to reflect equity.

In addition, as shown in Figure 16d, communities of different economic classes show steady performance in terms of their comprehensive green exposure levels across the built-up period, with one type of green exposure level increasing and then decreasing and two types of green exposure levels decreasing. The comprehensive green exposure level remains almost stable, reflecting the potential for the three types of green exposure levels to substitute for each other.

#### 4.4.2. Trend in Attainment Rates for Community Green Exposure 

**Figure 17:**
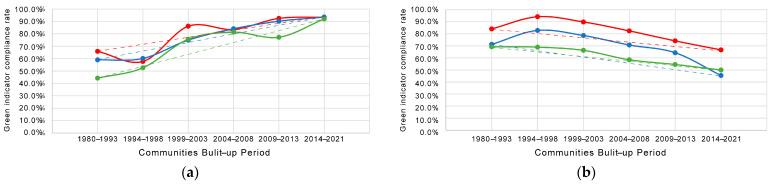

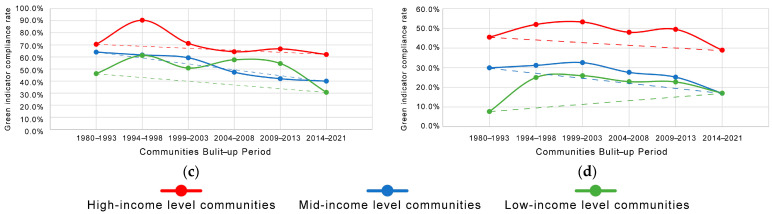
Meeting standards of green space levels in communities of different economic classes at different periods, in terms of (**a**) community green space ratio, (**b**) GVI, (**c**) park accessibility, and (**d**) comprehensive green exposure.

As shown in Figure 17a, while the attainment of green space ratios in early communities is low relative to the other two types of green space, the latest built-up communities (2014-) are already above 90%. Furthermore, the disparity in the attainment of green space ratios in communities of different economic classes is decreasing (Figure 17b,c). The attainment rates with GVI and accessibility standards for newer communities are declining; 50% and 30% of the latest built-up and low-income level communities have achieved GVI and accessibility standards, respectively.

Under the more stringent standards for comprehensive green exposure attainment rates, newer communities show a declining trend in attainment rates (Figure 17d). The earliest built-up and low-income level communities have very poor comprehensive green exposure attainment rates, with only 7.7% of communities meeting the standard from 1980 to 1993.

In the most recent (2014–) built-up communities, the attainment rates for the three green space levels and the comprehensive green exposure level have been almost equal between low-income and mid-income levels. Even in the communities built from 2004 to 2013, the attainment rates for park accessibility levels in low-income level communities have been significantly stronger than those in mid-income level communities.

#### 4.4.3. A Test of Variation in the Level of Community Green Exposure

Low-income level communities were used as the control group, and mid-income and high-income level communities were treated as the experimental group, respectively. The chi-square test in SPSS was used to explore whether there were significant differences in the attainment rates of green space ratio, GVI, accessibility, and comprehensive green exposure indicators between the groups of communities of different economic levels at different built-up periods.

The chi-square test results further confirm our view (Table 5). Overall, nearly half the periods (25/48, period of significant chi-square test/total period) have significantly higher attainment rates for green space levels in high- and mid-income level communities than in low-income level communities. The advantage of high- and mid-income level communities over low-income level communities in meeting green exposure levels is mainly in park accessibility (8/12), with a weaker advantage in community green space ratio (5/12) and GVI (5/12). Meanwhile, high-income level communities are significantly stronger than low-income level communities in terms of comprehensive green exposure levels in all periods. In contrast, mid-income level communities have a significant advantage over low-income level communities only from 1980 to 1993.

Finally, among the most recently built-up communities (2014–), only high-income level communities have a significant advantage over lower-income level communities in terms of park accessibility and comprehensive green exposure levels, with mid-income level communities no longer having a significant advantage. This is a phenomenon showing less social inequality, as access to qualifying levels of green space is not very different for all but the wealthiest.

### 4.5. The Relationship between Communities’ Built-Up Period and Green Exposure Levels

#### 4.5.1. The Reasons for Trends in Levels of Community Green Exposure

New communities are increasing their green space attainment rates based on the development trend of the built-up period. Still, GVI and park accessibility are generally lower than in previously built communities. These figures suggest the rising level of private greenery and the declining level of public greenery.

- Private green spaces.

The relationship between the timing of community completion and green space rates is explained in the previous Section 4.3.1. Currently, land use regulations in China require each new residential development to observe a specific green ratio before a plot is handed over to the market for auction, leading to a convergence in the level of green space ratio in later communities.

- Public green spaces.

On the one hand, current green space planning in China is based on a ‘top-down’ model, which, due to land constraints, does not complement public green space as easily as private community green space. Previously built-up communities already occupy good locations, and even in the same area new communities can only gradually move away from public green space. On the other hand, with the rapid growth of cities and the increasing size of built-up areas, the suburbanization of the residential population is a common phenomenon in large cities. However, urban green space is expanding slowly and has become a scarce resource, creating a spatial mismatch between its supply and demand [70].

#### 4.5.2. Typical Elements of the Green Space Trend: The Continued Dominance of High-Income Level Communities and the Catching Up of Low-Income Level Communities

With the level of private green space tending to be consistent across different economic classes and communities, the planning of public green space is a major concern for future green justice. Urban renewal and the suburbanization of green spaces have raised the level of green exposure in high-income and low-income level communities. This is why high-income level communities maintain the highest level of public green space most of the time, while low-income level communities can surpass the level of public green space of mid-income level communities during a certain period (2004 to 2013). Typical cases are as follows:

- Urban renewal in downtown communities.

A 74F super high-rise residential development located in the heart of Shenzhen’s downtown area, at Baishi Zhou, is surrounded by excellent greenery, with the OCT National Wetland Park to the east and the Dasha River Ecological Promenade to the west, both within a 10-min walk. This part of the residential block is planned to be built on the site of the old Baishi Zhou urban village redevelopment project. In urban regeneration, high-income inhabitants will be drawn to newly developed communities, while former low-income people will be screened out as part of the environmental gentrification process; approximately 150,000 low-income Baishi Zhou inhabitants have been displaced to low-quality communities on the edge of the city.

- The suburbanization of green space.

Communities within the center of Longgang district in the northeast of Shenzhen, which are mostly of low-income level, have benefited from the Greenland suburbanization strategy. Because the 2011 Shenzhen Universiade was held right in the center of the Longgang district, the big event led to massive investment in green infrastructure, including new parks and improved street greening. Public green spaces in the region have been significantly enhanced [71]. Regarding this, 0.2% of high-income level communities, 3.6% of mid-income level communities, and 22.3% of low-income level communities in the city are located in the central area of the Longgang district (the area of the Universiade venue), with more low-income groups benefiting from the green space dividends brought by the Universiade.

The 2008 Olympic Games in Beijing and the 2010 Youth Olympic Games in Nanjing were also major sporting events. However, the venues tend to be on the edge of the downtown area and not really in the city’s suburbs. The results did not enhance the green infrastructure for low-income groups [16,72].

## 5. Discussion

### 5.1. The Effects of Trends in Community Green Exposure

The impact of this trend on residents is not as bad as one might think. Firstly, the community green exposure attainment level trended consistently across economic classes (excluding low-income level communities in the period 1980–1993 to 1994–1998), reflecting social equity (Figure 17); private green exposure attainment levels, which are most frequently those in contact with residents [13], are rising. Moreover, the gap in the community green exposure attainment level between communities of different economic classes continues to narrow, even in the most recently built-up communities, with no significant gap (Figure 17a). Finally, on the one hand, there is no existence of substitutability between the three types of green spaces; on the other hand, our study confirms that the comprehensive green exposure level does not decrease.

The communities’ comprehensive green exposure level remains stable, suggesting some substitution between the various types of green space. Similarly, Zhang et al. [27] and Xiao et al. [13] identified some complementarity between private and public green spaces in their study of community green spaces in Beijing and Shanghai, China.

### 5.2. Strategies & Recommendations

Firstly, green space planning can be improved by considering communities’ green needs at different economic levels. Woo and Webster point out that traditional public goods provided by the government, such as urban green spaces, may be partially or completely replaced by club goods [73]. Therefore, from this perspective, we recommend that there is no need for the government to provide public green space for communities with high green space ratios for the time being. While the level of public green space in newer communities is declining, their level of private green space is generally up to standard. Some older communities have both private and public green spaces that are poor, so we recommend that the public green spaces in these communities be improved first. In this way, it will avoid the risk of wasting public resources through the over-provision of green space supply policies.

**Figure 18:**
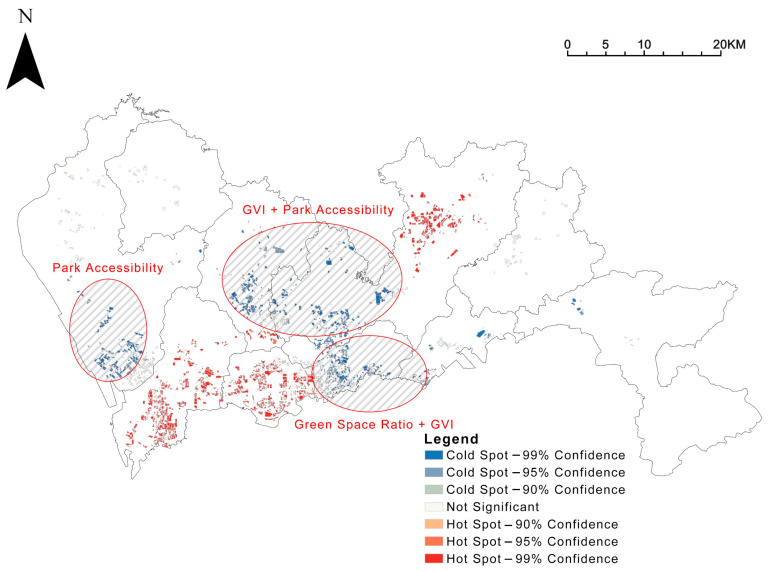
The distribution of communities that need to be supplemented with green space as a priority.

Secondly, we have studied spatial inequalities between the three types of green space in Shenzhen, which can help municipalities and planners to identify where and what they should invest more resources in to improve communities’ green exposure. In Shenzhen, this work has identified a gap: only 31.3% of communities meet all three basic objectives for green space simultaneously, with the vast majority (68.7%) of the remaining communities still not meeting it. Previously, we have added up the scores of the three green indicators for the community with the help of Standardization and Entropy Weighting and then analysed them based on the hotspot analysis (Figure 8). Further, we can mark three clusters on the Shenzhen map with poor performance on the combined greenness index score (Figure 18), which can be prioritized for further improvement. Taking into account the variability of greenery in the communities within each cluster, Figure 6 allows us to mark the content of their specific non-compliant green spaces.

In practical terms, community green spaces and parks are difficult to change; meanwhile, Xu et al. confirmed through VR experiments that streets with high GVI could replace places such as parks and green spaces as places for residents to release their stress [74]. Therefore, we suggest that these three clusters can first prioritize building on road infrastructure, enhancing three-dimensional greening along the streets to improve the street greening rate. At the same time, according to the first recommendation, the residential clusters in the Luohu district, where community green spaces are weakest, should prioritize placing public green facilities (street greening).

Finally, creating new parks is essential, but parks are the most difficult green spaces to add because of land and economic constraints. The ParkScore index by The Trust for Public Land (TPL) suggests that, in some cases, cities can improve park accessibility by adding new park entrances [75]. That is, in theory, the park could be considered to have entrances and exits in all (four) directions to increase accessibility.

**Figure 19:**
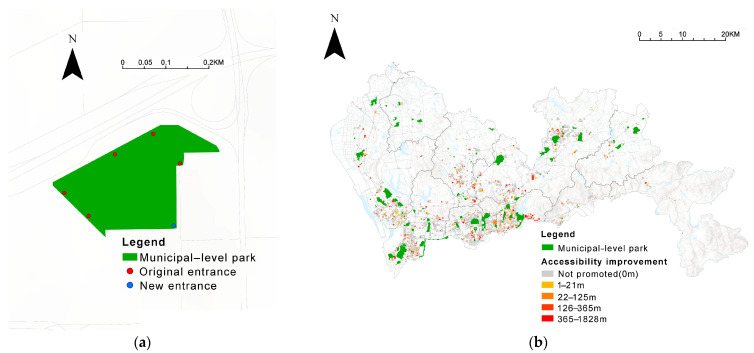
A programmer to enhance park accessibility: (**a**) added entrances and exits to the municipal park and (**b**) effect of the programmer.

Nearly 70% of Shenzhen’s municipal parks currently do not guarantee access in all four directions, and about 30% of municipal parks have access in only one to two directions, indicating that there is still much potential to improve accessibility. Specifically, as in Figure 19a, we artificially added an access point on the side of the municipal park that has no access direction, comparing how much the communities’ accessibility to the nearest municipal park has improved after the addition of the access point. The results are shown in Figure 19b, and 709 (21.6%) communities have improved park accessibility, reducing the distance by an average of 279.9 m; a further 94 communities have shifted from non-standard to standard park accessibility, increasing the original 33.9% combined park accessibility rate by approximately 2.9%.

It should be added that our emphasis is on the concept’s feasibility rather than the precise layout of each park entrance.

## 6. Conclusions

### 6.1. Differences in Community Green Exposure & Future Green Space Planning Strategies

The equitable distribution of green space is becoming more recognized as an issue of environmental inequality in China. This study uses multi-source big data to explore the green exposure inequality in built-up communities over time. The results show that: (1) green space ratio, GVI, accessibility, and comprehensive green exposure levels of Shenzhen’s communities are unevenly distributed among different economic classes, with lower-income level communities having poorer green exposure. (2) Compared to the old communities, the newly built communities have a higher green space ratio, lower GVI, and the comprehensive green exposure level scores remain stable. (3) Among the newly built-up communities, high-income level communities have a significant green space advantage over low-income level communities, but the difference between the mid-income and low-income level communities is not significant.

This study implies that, firstly, the differences in the level of community green space may be related to the uneven spatial distribution of green space resources in Shenzhen. Then, the differentiation of urban residents’ needs and abilities allows people with high economic attributes to live in communities with better levels of green space, building gaps in the level of community green space among different economic classes. Secondly, the level of private green space in newly built-up communities is increasing, while the level of public green space is decreasing. This trend is influenced mainly by residential design regulations and land constraints, but is not very bad, as green spaces are interchangeable. Finally, government planning for green facilities includes ‘green gentrification’ and green space planning derived from major (sporting) events. It has continued to give upscale communities a significant green space advantage and has helped close the green space gap between low and midscale communities.

For future green space planning, three policy implications are highlighted: (1) street greening should be enhanced in areas with weak performance in terms of comprehensive green exposure levels, (2) communities with poor private green spaces should be prioritized to enhance public green spaces around them, and (3) accessibility should be enhanced by ensuring that parks have entrances in every direction as far as possible.

### 6.2. Limitations

Our study has some limitations. Firstly, the GVI indicator is calculated from the greenery within 100 m of the community boundary, which is only relevant to those residents living directly within the community boundary. People whose daily activities pass through these areas also benefit from GVI. Secondly, park accessibility only takes into account the distance from the community to the nearest park and does not take into account the quality and quantity of parks within a certain range. Finally, some fee-paying parks were excluded from the study, but they may meet the daily use needs of some wealthy people. For example, residents of the community surrounding Happy Valley Park who have an annual pass can obtain free daily access to the fee-paying parks. The benefits of access to parks for the wealthy may have been underestimated.

## Figures and Tables

**Table 1:** Benefits of green space.

Type of Green Space	Description	The Role of Green Space	
		Common	Featured
Community gardens	Private greenery, which is closest to people and has the most frequent contact with them	Manufacturing oxygen [21], carbon fixation [22], improving air quality [23], drainage [24], heat absorption [25], and relaxing and fun [26] etc.	Increase opportunities for community activities to promote well-being [27], regulate community microclimate [28], and enhance community business value [29].
Street greenery	Public green spaces, with which people come into close contact during their daily travels		Noise absorption [30], sunshade [31], and raising the aesthetic level of urban places [32]. Improving the walkability of city streets and social behavior there [20].
Urban park	Public greenery, which is usually within a certain walking distance from people, is the most spacious and well-designed green space in the urban green space system		The space is relatively spacious and can provide entertainment value [33] to enhance well-being [34], and physical and mental health [35].

**Table 2:** Overview of green spaces.

Green Space Indicator	Proportion of Attainment	Mean-Benchmark	Coefficient of Variation	Mean-Green Exposure Level
Green space ratio	76.0%	0.907	0.347	33.2%
GVI	72.2%	0.943	0.318	31.2%
Park accessibility	56.9%	0.857	0.755	972.7 m

**Table 3:** Indicator weights for community comprehensive green exposure levels.

Type of Green Space	Entropy $\left(H_{i}\right)$	Weight $\left(W_{i}\right)$
Green space ratio	0.00342	0.37266
GVI	0.00132	0.14339
Park accessibility	0.00444	0.48395

**Table 4:** Downtown compared to non-downtown park levels.

Area	Park Area (ha)	Park Number (Individual)	City Built-Up Area (km^2^)	Number Ratio (Individual/km^2^)	Park Area Ratio (ha/km^2^)
Downtown	2722.57	109	166.02	0.66	16.40
Non-downtown	3487.28	288	813.98	0.35	4.28

**Table 5:** Differences in green space level attainment in communities of different economic classes at different periods.

Green Space Indicator	Group	Pearson Chi-Square in Different Period					
		1980–1993	1994–1998	1999–2003	2004–2008	2009–2013	2014–2021
	Reference group (low-income level communities)						
Green space ratio	mid-income level	3.851 *	2.132	0.037	0.492	7.264 *	0.230
	high-income level	4.512 *	0.377	6.808 **	0.251	8.231 *	0.159
GVI	mid-income level	0.087	10.841 **	11.801 **	6.123	2.187	0.530
	high-income level	2.889	12.858 **	30.790 **	20.216 **	7.637	5.576
Park accessibility	mid-income level	5.796 *	0.006	4.291 *	3.749 *	3.467 *	2.283
	high-income level	5.752 *	14.622 **	16.805 **	1.325	2.787	19.101 **
Comprehensive green exposure level	mid-income level	11.045 **	1.643	2.963	1.007	0.178	0
	high-income level	18.126 **	11.712 **	29.701 **	18.683 **	13.950 **	11.218 **

* and ** indicate *p*-values < 0.05 and <0.01, respectively.

## Data Availability

The data presented in this study are available on request from the corresponding author.
